# Kaposi's Sarcoma Associated-Herpes Virus (KSHV) Seroprevalence in Pregnant Women in South Africa

**DOI:** 10.1186/1750-9378-5-14

**Published:** 2010-08-31

**Authors:** Babatyi I Malope-Kgokong, Patrick MacPhail, Georgina Mbisa, Edith Ratshikhopha, Mhairi Maskew, Lara Stein, Freddy Sitas, Denise Whitby

**Affiliations:** 1 Clinical HIV Research Unit, Department of Medicine, Faculty of Health Sciences, University of the Witwatersrand, Johannesburg, South Africa; 2 Viral Oncology Section, AIDS and Cancer Virus Program, SAIC-Frederick, NCI-Frederick, Frederick MD, USA; 3 Cancer Epidemiology Research Group, National Health Laboratory Services, Johannesburg, South Africa; 4 Cancer Research Division, Cancer Council New South Wales, Australia

## Abstract

**Background:**

Factors previously associated with Kaposi's sarcoma-associated herpesvirus (KSHV) transmission in Africa include sexual, familial, and proximity to river water. We measured the seroprevalence of KSHV in relation to HIV, syphilis, and demographic factors among pregnant women attending public antenatal clinics in the Gauteng province of South Africa.

**Methods:**

We tested for antibodies to KSHV lytic K8.1 and latent Orf73 antigens in 1740 pregnant women attending antenatal clinics who contributed blood to the "National HIV and Syphilis Sero-Prevalence Survey - South Africa, 2001". Information on HIV and syphilis serology, age, education, residential area, gravidity, and parity was anonymously linked to evaluate risk factors for KSHV seropositivity. Clinics were grouped by municipality regions and their proximity to the two main river catchments defined.

**Results:**

KSHV seropositivity (reactive to either lytic K8.1 and latent Orf73) was nearly twice that of HIV (44.6% vs. 23.1%). HIV and syphilis seropositivity was 12.7% and 14.9% in women without KSHV, and 36.1% and 19.9% respectively in those with KSHV. Women who are KSHV seropositive were 4 times more likely to be HIV positive than those who were KSHV seronegative (AOR 4.1 95%CI: 3.4 - 5.7). Although, women with HIV infection were more likely to be syphilis seropositive (AOR 1.8 95%CI: 1.3 - 2.4), no association between KSHV and syphilis seropositivity was observed. Those with higher levels of education had lower levels of KSHV seropositivity compared to those with lower education levels. KSHV seropositivity showed a heterogeneous pattern of prevalence in some localities.

**Conclusions:**

The association between KSHV and HIV seropositivity and a lack of common association with syphilis, suggests that KSHV transmission may involve geographical and cultural factors other than sexual transmission.

## Background

Kaposi Sarcoma-associated herpesvirus (KSHV), also known as Human Herpesvirus 8 (HHV-8) is the causative agent of Kaposi's sarcoma (KS) [1,2], and is associated with primary effusion lymphoma (PEL) [3] and multicentric Castleman's disease [4]. Prevalence of KSHV is elevated in Mediterranean populations [5] and high in sub-Saharan Africa [6-8]. Unlike in the United States and Northern Europe, where KSHV is common mostly in men who have sex with men (MSM), in these endemic regions KS and KSHV affect the general population and it is increasingly apparent that non-sexual modes of transmission play a significant role in the maintenance and spread of KSHV [9,10]

The biological, social and environmental factors involved in non-sexual horizontal transmission of KSHV are still largely unknown. The HIV epidemic has had a profound effect on the rate of KS development in Africa. In South Africa, HIV co-infection is associated with up to 50 fold increases in risk for developing KS [11]. The role of HIV as a risk factor for KSHV infection in South Africa is unclear; some reports show a strong association whereas others show none [9,12]. Several studies that show a strong association between HIV and KSHV infection fail to show a similar strong association with other sexually transmitted infections that are clearly associated with HIV infection [9,13]. Evidence against sexual transmission of KSHV in heterosexual populations continues to emerge [12,14-16]. KSHV infection has been associated with sources of drinking water and with living in close proximity to rivers or streams [17,18]. However, the role of vectors and environmental factors in KSHV endemic countries is a topic of ongoing study [19,20].

HIV seroprevalence in pregnant women attending public sector antenatal clinics has been used as a reliable gauge of the South African HIV epidemic [21,22]. Understanding KSHV infection patterns in this group of women will provide a reasonable and comparable estimate of its impact in the same communities. This study aims to examine the seroprevalence of KSHV in pregnant women attending antenatal clinics and to identify the risk for KSHV infection in relation to already collected information on socio-demographic and geographical factors, HIV and syphilis serology.

## Materials and methods

### Study Patients

**Figure 1:**
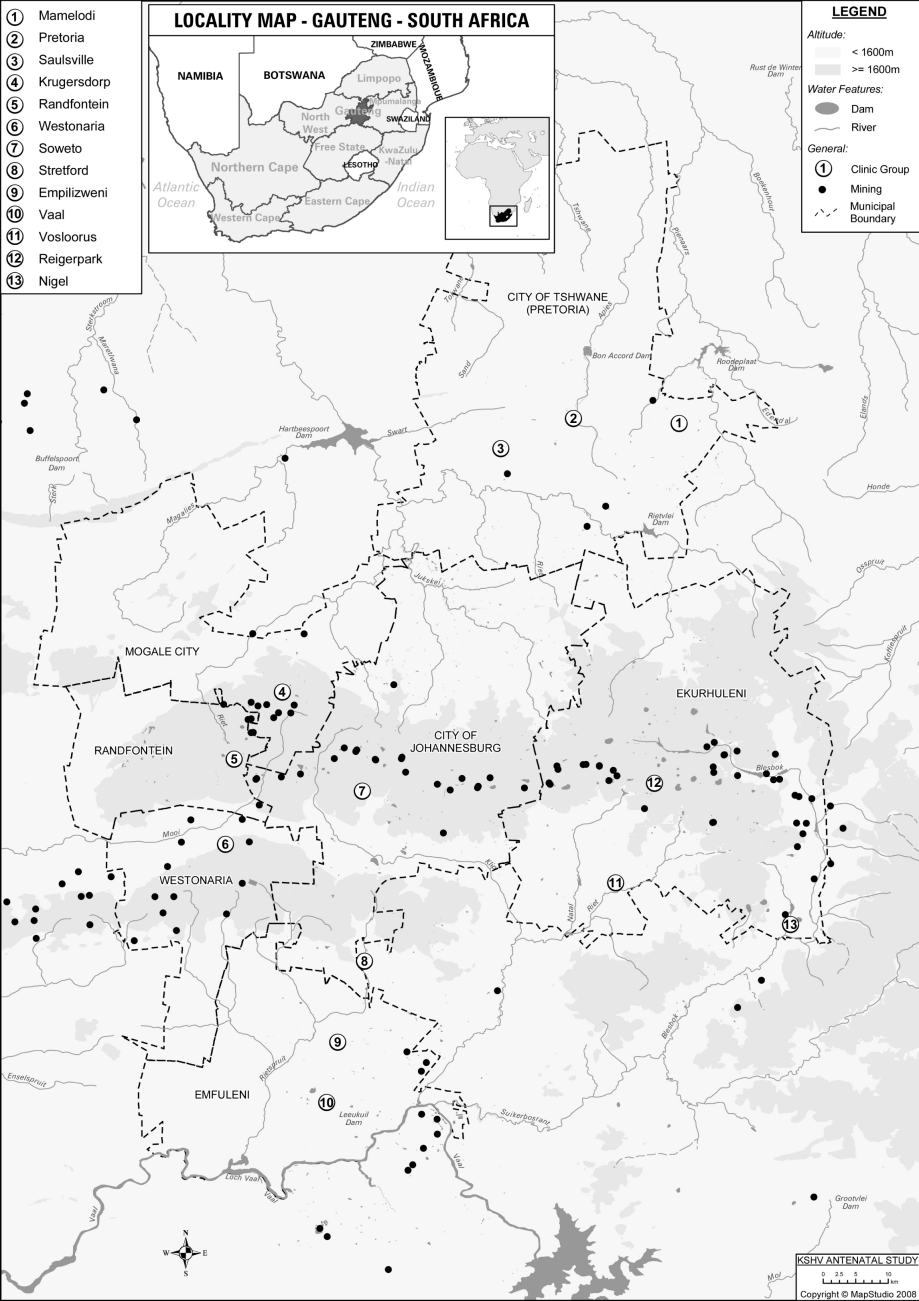
**Map of Gauteng province showing the locations of the ante-natal clinics**. Locations of ante-natal clinics from which study participants were recruited are shown according to the embedded legend. Altitude, water features and municipal boundaries are also shown according to the embedded legend.

This cross sectional study was conducted among 1740 black pregnant women attending public sector antenatal clinics in Gauteng province, South Africa. Women were recruited for the study at their first visit to the clinic during their current pregnancy. The women formed part of a national HIV and sexually transmitted infections (STI) study conducted by the National Department of Health in 2001. A total of 37 clinics within the Gauteng Province formed part of this study. Subjects were then divided into five groups according to the municipalities in which the antenatal clinics were located. These were: East Rand, Soweto, Pretoria, Vaal Triangle and West Rand (Figure 1). Gauteng province is the smallest but second most populated province in South Africa, occupying a total area of 17 010 km^2^. It is mostly urbanized and is home to over 9.6 million people, over a fifth of the national population [23]. The East and West Rand regions are dominated by mining (Figure 1), while the Vaal Triangle contains mainly manufacturing sectors with a mix of agriculture, heavy and petrochemical industries. Tshwane contains light industrial and residential areas, while Soweto is largely residential with residents working mainly in Central Johannesburg and the West Rand. Information on age, education level, parity and gravidity of subjects was collected as part of the original study. Geographic information on river catchments in the areas was deduced from the location of the different clinics. Ethics approval was granted by the University of the Witwatersrand Research Ethics Committee (Medical).

### Laboratory Analysis

Laboratory diagnosis of HIV and syphilis was done by the National Health Laboratory Services (NHLS), South Africa. HIV antibodies were determined using an Abbot Axysm System for HIV-1/HIV-2 ELISA assays (Abbott Laboratories, Diagnostics Division, Abbott Park, Illinois) and syphilis antibodies were determined using a non-treponemal carbon antigen test. Leftover sera were stored at -20°C before being shipped to the Viral Oncology Section, AIDS and Cancer Virus Program, Science Applications International Corporation (SAIC), National Cancer Institute (NCI), Maryland, for determination of antibodies to lytic K8.1 and latent open reading frame (Orf) 73 KSHV antigens. In-house assays for detection of antibodies to lytic K8.1 and latent Orf73 KSHV antigens were used as detailed previously [12,23]. Seropositivity to KSHV was further defined when subjects tested seropositive to either lytic KSHV K8.1 or latent KSHV Orf73 antibodies. Available information on age, education level, parity, gravidity, laboratory HIV and syphilis results was anonymously linked to the KSHV data using unique participant identification numbers.

### Statistical Analysis

Descriptive statistical analysis and measures of effect were done using SAS 9.1 (SAS Institute Inc, Cary, NC, USA.). The Kappa coefficient (κ) was calculated to determine concordance between antibodies against the lytic K8.1 and latent Orf73 antigens. Logarithmic transformation of the antibody titres (expressed as optical densities) for lytic K8.1 and latent Orf73 allowed for the use of standard parametric statistical methods and results are expressed as the geometric mean (μg) and standard deviation range (σg). Comparisons of means were done using Bonferroni adjusted student t-test between groups or Analysis of Variance (ANOVA) amongst multiple groups. Trends were measured using the Cochran-Armitage Trend Test. We calculated prevalence odds ratios (PORs) and 95% Wald confidence intervals (CIs) for KSHV seropositivity using logistic regression. In a multivariate model PORs were adjusted for age group (≤ 20, 21-25 26-30, ≥ 31), education level in years (< 2 years, 2-5 years, 6-12 years and >12 years (post matriculation/secondary school) of formal education), municipal region (East Rand, Soweto, Pretoria, Vaal Triangle and West Rand), syphilis seropositivity. Chi-square (χ) tests for binary measures, trend and homogeneity were calculated and two-sided p-values were used to measure the significance of the associations before and after adjustment for other covariates.

## Results

The mean (± SD) age of all 1740 pregnant women included in the study was 26.0 (± 6.2) years. Age was similar amongst the following four municipal regions: Ekurhuleni (26.5 (± 6.3)), Tshwane (25.8 (± 6.1)), West Rand (26.4 (± 6.1)) and Emfuleni (25.9 (± 6.3)) (Table 1). However, pregnant women attending clinics in Soweto (24.9 (± 5.8)), were significantly younger than those from the West Rand and Ekurhuleni municipalities (p = 0.043).

**Table 1:** Lytic and latent KSHV antibody levels and Odds Ratios (OR's) for seropositivity by municipal region, HIV and syphilis status and education

			**Lytic K8.1**			**Latent Orf 73**		
			
	**Total (n)**	**Age Mean (± SD)**	**Total Positive (n) (% Positive)**	**Antibody level μg(SD range)**	**Adjusted OR (95%CI)**	**Total Positive (n)**	Antibody level μg(SD range)*	**Adjusted OR (95%CI)**
		
**All Subjects**	**1740**	26.0(6.2)	568(32.6)	0.77(0.31 -1.38)	-	568(32.6)	0.40(0.11-0.76)	-
**Municipality Region**								
Soweto	293	24.9(5.8)^ab^	76(25.9)	0.71(0.27-1.29)^b^	1	79(27.0)	0.37(0.09-0.73)	1
Ekurhuleni	567	26.5(6.3)^a^	204(36.0)	0.79(0.30 - 1.46)	1.7(1.2 - 2.3)*	195(34.4)	0.40(0.11-0.77)	1.5(1.0 - 2.1)
Emfuleni	330	25.9(6.3)	107(32.4)	0.75(0.32-1.32)	1.5(1.0 - 2.1)*	118(35.8)	0.40(0.13-0.74)	1.7(1.2 - 2.4)*
Tshwane	207	25.8(6.1)	53(25.6)	0.71(0.32 -1.21)^a^	1.1(0.7 - 1.7)	52(25.1)	0.36(0.10-0.68)	1.0(0.7 - 1.6)
West Rand	343	26.4 (6.1)^b^	128(37.3)	0.84(0.37-1.49)^ab^	1.6(1.1 - 2.3)*	124(36.2)	0.43(0.13-0.82)	1.5(1.0 - 2.2) *
***P*_*4df*_**		*0.0063*		*0.0069*	*0.0233*		*0.0675*	*0.0185*
**Education Level**								
< 2 years	117	28.5 (7.9)^a^	46(39.3)	0.90(1.67-0.35)^a^	1	47(40.2)	0.45(0.12-0.90)	1
2 - 5 years	513	26.5 (6.8)^b^	193(37.6)	0.82(0.32-1.48)^b^	0.8 (0.5 - 1.2)	176(34.3)	0.40(0.11-0.85)	0.6(0.4 - 1.0)
6 - 12 years	119	26.4 (5.6)	35(29.4)	0.75(0.30-1.36)	0.4(0.3 - 0.8)*	37(31.1)	0.42(0.14-0.80)	0.5(0.3 - 0.9) *
Post Matric	959	25.4 (5.5)^ab^	286(29.8)	0.73(0.31-1.30)^ab^	0.6(0.4 - 0.8)*	300(31.3)	0.42(0.11-0.72)	0.6(0.4 - 0.9) *
***P*_*3df*_**		*0.042*		*0.0019*	*0.002*		*0.0874*	*0.0426*
**HIV Infection**								
Negative	1338	26.2 (6.4)	345(25.8)	0.70(0.28-1.27)	1	341(25.5)	0.36(0.09-0.68)	1
Positive	402	25.3 (5.4)	223(55.5)	1.00(0.50-1.69)	3.7(2.9 - 4.7) *	227(56.5)	0.54(0.21-0.98)	3.8(3.0 - 4.8) *
***P*_*1df*_**		*0.011*		***< 0.0001***	***< 0.0001***		* < 0.0001*	*< 0.0001*
**Syphilis Infection**								
Negative	1442	25.9 (6.2)	461(32.0)	0.77(0.31-1.38)	1	454(31.5)	0.39(0.10-0.75)	1
Positive	298	26.4 (5.8)	107(35.9)	0.79(0.34-1.38)	1.0 (0.7 - 1.3)	114(38.3)	0.43(0.15-0.81)	1.2 (0.9 - 1.6)
***P*_*1df*_**		*0.24*		*0.55*	*0.8924*		*0.087*	*0.2395*

^#^μg(SD range) geometric mean and standard deviation range. Means with the same letter are significantly different (p, 0.05). * Statistically significant Odds Ratios.

### Lytic and latent KSHV Serology

One third (568) of all the subjects were seropositive to lytic K8.1 antibodies and another overlapping third (568) were seropositive to latent Orf73 antibodies (Table 1). Overall, 776 (44.6%) subjects had antibodies to either lytic K8.1 only (208), latent Orf73 only (208) or both (360) and were considered to be KSHV seropositive. There was moderate concordance in seropositivity between the two (K8.1 and Orf73) assays (κ = 0.46 95% CI: 0.41 - 0.50) consistent with previous studies[12,24].

The geometric mean (μ_g_) of optical density for lytic K8.1 was 0.77((σ_g _0.31 - 1.38). The mean lytic K8.1 antibody level, expressed as optical densities, were significantly heterogeneous between municipal regions (*P_4df _= 0.0069*) and education levels (*P_4df _= 0.0019*) (Table 1). Women in Tshwane and Soweto had lower lytic K8.1 optical densities than those from the West Rand (μ_g _(σ_g_):0.71(0.32 - 1.21) and 0.71(0.27 - 1.29), vs. 0.84(0.37 - 1.49), respectively (*P*_*4df *_= 0.0069)]. The μ_g _(σ_g_) for latent orf73 was 0.40 (0.11 - 0.76) and did not differ significantly between regions (*P_4df _= 0.0675*) or education levels (*P_3df _= 0.0874*). HIV positive women had significantly higher lytic and latent KSHV antibody levels than HIV negative women (μ_g _(σ_g_): 1.0(0.50 - 1.69) vs. 0.70(0.28 - 1.27), (*P_1df _< 0.0001*) and 0.54(0.21 - 0.98), vs. 0.36(0.09 - 0.68), respectively) (*P_1df _< 0.0001*) (Table 1).

### Risk Factors for KSHV

#### Age, Education and selected characteristics

Seropositivity to KSHV in these women in their reproductive years was not associated with age (*P_trend _= 0.5988*) (Table 2). While older age appeared to be protective against HIV infection, this relationship was not seen in the adjusted model. An inverse trend for KSHV was noted with higher education levels (*P_trend _= 0.0015*) being protective against KSHV but not HIV infection. More than half (53.0%) of the 117 women with no formal education (< 2 years) were seropositive to KSHV decreasing to 41.7% in the 959 women with the highest high school matriculation certificate (*P_3df _= 0.008*). The number of pregnancies and live births had no effect on KSHV seropositivity. (Table 2).

**Table 2:** Factors affecting the odds ratios (OR) for KSHV and HIV in pregnant women attending antenatal public clinics in Gauteng province of South Africa.

		**KSHV Seropositivity**			**HIV Seropositivity**		
		
	**Total (n)**	**Prevalence** **Total n(%)**	**Unadjusted OR^# ^(95%CI)**	**Adjusted OR (95%CI)**	**Prevalence** **Total n(%)**	**Unadjusted OR (95%CI)**	**Adjusted OR^# ^(95%CI)**
		
**All Subjects**	**1740**	**776(44.6%)**	**-**	**-**	402(23.1%)	-	-
**Age Group**							
≤ 20	362	154 (42.4)	1	1	87(24.0%)	1	1
21 - 25	533	246 (46.2)	1.2 (0.9 - 1.5)	1.2 (0.9 - 1.7)	136(25.5%)	1.1 (0.8 - 1.5)	1.1 (0.7 - 1.5)
26 - 30	438	192 (43.8)	1.1 (0.8 - 1.4)	1.1 (0.8 - 1.5)	108(24.7%)	1.0 (0.7 - 1.5)	1.0 (0.7 - 1.5)
≥ 31	407	184 (45.2)	1.1 (0.8 - 1.5)	1.2 (0.9 - 1.6)	71(17.4%)	0.7 (0.4 - 0.9)*	0.7 (0.5 - 1.0)*
*P*_*3df*_			*0.6240*	*0.4830*		*0.0286*	*0.0373*
**Municipality Region**							
Soweto	293	105(35.4)	1	1	72(24.6%)	1	1
Ekurhuleni	567	274 (48.3)	1.6 (1.3 - 2.2)*	1.8 (1.3 - 2.4) *	147(25.9%)	1.0 (0.7 - 1.5)	1.1 (0.7 - 1.6)
Emfuleni	330	152 (46.1)	1.5 (1.1 - 2.1) *	1.8 (1.1 - 2.6) *	63(19.1%)	0.8 (0.5 - 1.1)	0.7 (0.5 - 1.1)
Tshwane	207	77 (37.2)	1.1 (0.7 - 1.5)	1.3 (0.5 - 1.9)	29(14.0%)	0.5 (0.3 - 0.8)*	0.5 (0.3 - 0.8)*
West Rand	343	168 (49.0)	1.7 (1.2 - 2.4) *	1.7 (1.1 - 2.5) *	91(22.6%)	1.1 (0.8 - 1.6)	1.1 (0.7 - 1.7)
***P***_*4df*_			*0.0015*	*0.0095*		*0.0017*	*0.0013*
**Education Level**							
< 2 years	117	62 (53.0)	1	1	17(14.5%)	1	1
2- 5 years	513	253 (49.3)	0.9 (0.6 - 1.3)	0.7 (0.5 - 1.1)	119(23.2%)	1.3 (0.8 - 2.1)	1.4 (0.8 - 2.6)
6 - 12 years	119	51 (42.9)	0.7 (0.4 - 1.1)	0.5 (0.3 - 0.8)*	31(26.0%)	1.6 (0.8 - 3.1)	1.4 (0.7 - 2.8)
Post Matric	959	400 (41.7)	0.6(0.4 - 0.9)*	0.5 (0.4 - 0.8)*	228(23.8%)	1.4 (0.9 - 2.1)	1.6 (0.9 - 2.8)
*P*_*3df*_			*0.0080*	*0.0034*		*0.1645*	*0.3437*
**Gravidity Group**							
1	631	269 (42.6)	1	-	144(22.8%)	1	-
2	481	223 (46.4)	1.2 (0.9 - 1.5)	-	129(26.8%)	1.2 (1.0 - 1.6)*	-
≥2	532	246 (46.2)	1.2 (0.9 - 1.5)	-	106(28.0%)	0.8 (0.6 - 1.1)	-
***P***_*2df*_			*0.2863*			*0.0284*	
**Parity group**							
0	649	279 (43.0)	1	-	149(23.0%)	1	-
1	487	223(45.8)	1.1 (0.9 - 1.5)	-	129(26.5%)	1.2 (0.9 - 1.6)	-
> 2	506	235(46.4)	1.2 (0.9 - 1.5)	-	56(20.1%)	0.9 (0.6 - 1.1)	-
***P***_*2df*_			*0.3594*			*0.0263*	
**River Catchment**							
North	279	109 (39.1)	1	-	48(17.2%)	1	-
South	1461	667 (45.6)	0.8 (0.4 - 1.5)	-	354(24.2%)	0.6 (0.4 - 0.9)*	-
***P***_*1df*_			*0.4312*			*0.0075*	
**Syphilis**							
Non-reactive	1440	621 (43.1)	1	1	303(21.0%)	1	1
Reactive	298	154 (51.7)	1.4 (1.1 - 1.8)*	1.2 (0.9 - 1.6)	97(32.5%)	1.8 (1.4 - 2.4)*	1.9 (1.4 - 2.5)*
***P***_*1df*_			0.0028	0.1168		*< 0.0001*	*< 0.0001*
**HIV infection**							
Negative	1338	496 (37.1)	1	1		-	-
Positive	402	280 (69.7)	3.9 (3.1 - 5.0)*	4.1 (3.1 - 5.2)*		-	-
*P*_*1df*_			*< 0.0001*	*< 0.0001*			

^# ^Adjusted for age group, education levels, municipal region, syphilis seropositivity and/or HIV status. *Statistically significant Odds Ratios.

#### KSHV, HIV and Syphilis serology

The overall seroprevalence of HIV and syphilis, in pregnant women across all the clinics was 23.1% and 17.1%, respectively. Overall, 34.6% of all the women had either HIV or syphilis but only 6% were infected with both. Seropositivity to KSHV was 69.7% in HIV infected women and 37.1% in uninfected women (p < 0.0001) (Table 2). Pregnant women with syphilis also had higher KSHV seroprevalences than those without (51.7% vs 43.1% respectively; p = 0.0028). The prevalence of HIV and syphilis were 12.7% and 14.9% in pregnant women without KSHV, and 36.1 and 19.9% in those with positive KSHV serology. The risk for KSHV was 4.1 fold higher in those with HIV infection (4.1 95% CI: 3.1 - 5.2). However, KSHV was not associated with syphilis (AOR 1.2 95% CI: 0.9 - 1.6) (Table 2). KSHV antibody levels were higher in women with HIV but not in women with syphilis (Table 1)

#### Geographic factors and KSHV serology

Seropositivity to KSHV was significantly different amongst the municipal regions, ranging from 35.4% in Tshwane to 49.0% in the West Rand (*P_4df _= 0.0015*) (Table 2). Risks of KSHV infection was higher in all pregnant women attending clinics in Ekurhuleni and Emfuleni and the West Rand regions, compared to women attending clinics in Soweto (AOR 1.8 95% CI: 1.3 - 2.4; 1.8 95% CI: 1.1 - 2.6; and 1.7 95% CI: 1.1 - 2.5) (Table 2). While the risk for KSHV was similar between Soweto and Tshwane, women in the Tshwane region seem to be at a low risk for HIV infection compared to those from Soweto (AOR 0.5 95%CI: 0.3 - 0.8) (Table 2).

To further explore this geographic variation in KSHV serology within this region, we looked at the river catchments within the different regions. It was clear that there were two main river catchment systems draining south and north (Figure 1). KSHV seroprevalence was higher in the areas with the southern river drainage (45.6%) than those to the north (39.0%), however this difference was not significant (p_1df _= 0.4, Table 2).

## Discussion

Although HIV prevalence in South African women attending public sector antenatal clinics is well described [21], little is known about their KSHV status. In the sub-Saharan African setting it is now apparent that KSHV is an endemic infection affecting both children and adults [10,12,25,26] and that HIV is a significant co-factor in the pathogenesis of Kaposi's sarcoma [2,27,28]. In addition to studies suggesting sexual transmission of KSHV [26] and among HIV negative individuals [29], other studies on the epidemiology of KSHV from endemic African and Mediterranean countries have also established that the virus is transmitted via non-sexual routes [9,10,12,30]. Our findings provide further evidence for non-sexual horizontal transmission of KSHV, likely via saliva. However, risk factors for KSHV infection, the exact mode of KSHV transmission and other epidemiological co-factors that may promote KSHV infection need further elucidation.

In this study, KSHV seroprevalence in pregnant women attending antenatal clinics in the Gauteng province of South Africa was very high (45%) and nearly double that of HIV infection (23%). This further confirms that KSHV infection is very common in Southern African women consistent with previous reports where reported prevalence ranged from 30% - 46% [6,12,26]. HIV was strongly associated with increased risk for KSHV seropositivity as well as the risk of having both lytic and latent antibodies, as we have previously reported in a study of mothers and children from throughout South Africa [12]. However, we have also previously demonstrated in a mining community (Carletonville) with very high prevalence of both HIV and KSHV, that KSHV was not associated with HIV infection nor was it associated with other sexually transmitted infections [9]. The reasons for this discrepancy is unclear but may relate to the very high HIV prevalence in the Carletonville study population[9]. None of these studies found any association between KSHV infection and other STD including syphilis. In this study HIV positive subjects had significantly higher KSHV antibody levels than their HIV negative counterparts, an association not noted for syphilis. Higher antibody levels in HIV positive subjects may reflect poor immune control of KSHV resulting in more frequent reactivation.

The lack of association between KSHV and age noted in this study may be attributed to the narrow age range studied (mean (± SD) 25.9 (6.2)). In this study, pregnant women with a higher level of education had lower rates of KSHV infection. The association between KSHV and increasing education has been shown in other studies [29]. Education is often reported to be a surrogate marker of socio-economic status. implying that those with lower socio-economic status are at a higher risk for KSHV infection, consistent with previous reports [18,29].

We observed significant variation in the prevalence of KSHV between the regions within the Gauteng province of South Africa which remained after adjustment for several factors including age, HIV, syphilis status and education (Table 2). This is the first study in sub-Saharan Africa to demonstrate such geographical variation within a province. In the apartheid era, most South African townships were segregated according to ethnic and, therefore, geographic origin of the residents. It follows that some of this variation may be explained by differences in cultural practices.

Living in close proximity to rivers and streams has previously been associated with increased risk for KSHV [17]. We did not have information on place of residence for the women in our study, only the clinic that they attended. Therefore, we were unable to test this hypothesis. Further studies are warranted to clarify the nature of the geographical variation in KSHV prevalence observed in this study.

Women attending routine ante-natal clinics are frequently used in surveys of HIV prevalence in South Africa and other African countries. Assessing KSHV prevalence in this population therefore allows for valid comparisons between this study and future studies in other parts of South Africa or other African countries. This study adds to findings in South Africa and other African countries [31] which suggest a lack of evidence for sexual transmission of KSHV in heterosexual African populations. Cross-sectional studies of KSHV and other serology, while providing important information on risk factors for KSHV transmission, have limitations. A longitudinal study of KSHV transmission in South Africa is needed as well as studies aiming to identify geographical, cultural and/or lifestyle and environmental factors that may predispose people to KSHV infection.

## Competing interests

The authors declare that they have no competing interests

## Authors' contributions

BIM-K performed the statistical analysis, wrote the first draft of the manuscript and participated in the design of the study, PM and MM supervised the statistical analysis and contributed to the writing of the manuscript, GM performed the KSHV serology, FS conceived the study and with LS and ER, participated in the design of the study, the statistical analysis and contributed to the writing of the manuscript, DW participated in the design of the study, supervised the KSHV serology and contributed to the writing of the manuscript. All authors read and approved the final manuscript.
